# Functional Modification and Applications of Rice Starch Emulsion Systems Based on Interfacial Engineering

**DOI:** 10.3390/foods14132228

**Published:** 2025-06-24

**Authors:** Pingyuan Ge, Ye Tian, Heng Yan, Qingqing Li, Tianle Yao, Jie Yao, Liuyu Xiao, Meng Zhu, Yu Han

**Affiliations:** 1 National “111” Center for Cellular Regulation and Molecular Pharmaceutics, Hubei University of Technology, Wuhan 430068, China; 2211641125@hbut.edu.cn; 2 Department of Biological Science and Technology, Wuhan Bioengineering Institute, Wuhan 430415, China; tianye85@whsw.edu.cn; 3 Hubei Provincial Institute for Food Supervision and Test, Wuhan 430075, China; hbqtyan@163.com; 4 College of Food Science & Technology, Huazhong Agricultural University, Wuhan 430070, China; 5 Hubei Key Laboratory of Resource Utilization and Quality Control of Characteristic Crops, College of Life Science and Technology, Hubei Engineering University, Xiaogan 432000, China; 13135631690@163.com (Q.L.); ytl20031225@163.com (T.Y.); 13477171153@163.com (J.Y.); x372255967@163.com (L.X.)

**Keywords:** rice starch, emulsion technology, interfacial properties, Pickering emulsion, microcapsules

## Abstract

Rice starch, as one of the most abundant and renewable polysaccharide resources in nature, holds great potential for applications in the food, pharmaceutical, and industrial fields due to its wide availability, low cost, and biodegradability. However, its inherent limitations—such as susceptibility to retrogradation and poor emulsifying capacity—have hindered its development into high-value-added products. Emulsion technology presents a promising strategy to overcome these challenges by constructing stable oil–water interfacial systems using various stabilizers. This review highlights recent advances in the functional modification of rice starch through emulsion-based techniques, with a particular focus on four key approaches: polysaccharide–protein complexation, chemical and physical modifications, Pickering emulsions, and microcapsule formation. These strategies significantly improve the emulsifying ability of rice starch, inhibit retrogradation, and expand its potential applications in sustained drug delivery, functional foods, and intelligent packaging. Overall, interfacial engineering of rice starch offers an innovative and effective pathway for its high-value utilization, demonstrating substantial promise for future industrial applications.

## 1. Introduction

With the growing global emphasis on sustainable development and green chemistry, the development and functionalization of natural polymer materials has emerged as a prominent research focus. Rice (*Oryza sativa* L.), one of the most widely cultivated crops globally, had a planting area of 160 million hectares in 2023, spanning more than 120 countries across Asia, Africa, and South America, with an annual yield exceeding 780 million tons. Among this yield, 15–20% of rice-particularly low-eating-quality varieties such as IR64 and Thailand’s RD43 is directly utilized for industrial starch extraction, while by- products such as broken rice and rice bran achieve a utilization rate of 60–70% [1,2,3].

As the most abundant renewable polysaccharide resource, starch plays a crucial role in the food, pharmaceutical, and industrial sectors due to its broad availability and biodegradability. Global rice starch production rose from 8.9 million tons in 2021 to 9.5 million tons in 2023, with Asia accounting for 78% of output (including 38% from China and 25% from Thailand) [4,5]. Leveraging the large-scale use of low-grade rice and its by-products, rice starch production costs are 12–15% lower per ton compared to corn starch.

Despite these advantages, native starch suffers from inherent limitations—such as poor emulsification, susceptibility to retrogradation, and sensitivity to environmental conditions—which hinder its application in high-end products. For instance, although high-amylose rice starch exhibits 20–25% greater film-forming strength and a higher gelatinization temperature (75–80 °C) compared to corn starch, its lack of interfacial activity makes it difficult to stabilize emulsions. Moreover, the retrogradation of amylose at low temperatures shortens product shelf life, and extreme pH or thermal environments can easily compromise starch’s structural stability [6].

While emulsion technology has shown promise in overcoming these limitations for starches in general, a critical gap exists in the synthesis and critical evaluation of its specific application and impact on rice starch functionality. Existing research often focuses on specific techniques (Pickering emulsions or chemical modification) or broader starch categories, lacking a comprehensive review dedicated to the multifaceted role of emulsion strategies—including polysaccharide–protein complexation, modification, Pickering emulsions, and microencapsulation—in enhancing rice starch properties and their collective industrial viability.

In recent years, emulsion technology has offered a promising approach to enhance starch functionality by constructing oil–water dispersion systems using interfacial stabilizers. This technique not only imparts interfacial activity to starch through chemical modifications (e.g., esterification and etherification), but also inhibits retrogradation via physical oil-phase barriers [7,8]. Additionally, cross-linking and composite encapsulation strategies have greatly improved starch’s environmental stability, making it suitable for challenging applications such as acidic beverages and thermally sterilized foods. More importantly, emulsion technology has expanded the functional versatility of starch by enabling the incorporation of active ingredients for targeted delivery and the development of intelligent, stimuli-responsive materials.

The aim of this study is to comprehensively analyze the role of emulsion technology in upgrading the functionality of rice starch. Four key application strategies—polysaccharide–protein complexation, starch modification, Pickering emulsions, and microcapsule fabrication—are discussed, alongside their industrialization prospects.

## 2. Physicochemical Properties and Limitations of Rice Starch

Starch is one of the most abundant and renewable polysaccharides in nature. Its structural diversity and wide availability make it valuable in food, pharmaceutical, industrial, and emerging fields [9]. Whether as a natural energy storage material or a functional substance enhanced through modification, the performance and application of starch are inherently determined by its physicochemical properties [10]. Compared with common starches like corn or potato, rice starch shows unique advantages such as small granule size, environmental friendliness, and ease of molecular tuning, providing great potential in applications demanding high precision and sustainability.

### 2.1. Classification of Starch

Starch is a macromolecule composed of glucose units joined by α-1,4 and α-1,6 glycosidic bonds, mainly including amylose (linear) and amylopectin (branched) [11,12,13]. Their ratio significantly affects starch properties like gelatinization, retrogradation, and film formation.

By origin, starch is classified into seed, tuber, and rhizome starches. Seed starches (e.g., corn, wheat, rice) have polygonal/spherical granules and varied amylose content. Tuber starches (e.g., potato, cassava) have larger, smoother granules with high amylopectin content (~80%), making them ideal for film-forming applications. Rhizome starches (e.g., sweet potato) gelatinize into high-viscosity pastes, often used for thickening (Table 1) [12,13].

By structure, high-amylose starch (>50% amylose) has strong film-forming ability and is suitable for biodegradable materials [12], while high-amylopectin starch (>80%) shows excellent gelling and clarity, ideal for food thickeners [13]. Though high-amylose rice starch currently holds <1% market share in degradable packaging, its market is growing at over 20% annually, showing future potential through scalable production [14,15,16].

Starch can also be classified by function into normal, waxy, and resistant types. Normal starch (e.g., potato) is widely used in conventional food processing [17]. Waxy starch, nearly amylose-free, provides strong freeze–thaw stability, useful in frozen foods [18]. Resistant starch, such as green banana starch, resists enzymatic digestion and supports low-GI food development [19,20].

**Table 1:** Common starch classification table.

Classification Criteria	Type	Representative Starch	Granule Morphology	Amylose/Amylopectin Ratio	Gelatinization Temperature	Characteristics and Applications	Emulsifying Performance (Emulsifying Activity Index)	References
By source	Seed starch	Corn starch	Polygonal or spherical	Direct links account for approximately 25%	-	It has strong film-forming properties and is used in degradable packaging materials.	10–30 m^2^/g	[11]
		Wheat starch			-	It has strong gelling power and is suitable for traditional noodles.	15–35 m^2^/g	[11]
		Wheat starch		Direct chain: 15–30%	60–70 °C	The viscosity is moderate after gelatinization and it is prone to aging.	10–25 m^2^/g	[11]
	Tuber starch	Potato starch	Large particles with a smooth surface	Branched chains >80%	58–65 °C	It has excellent film-forming properties and transparency, and is used in degradable packaging materials.	20–40 m^2^/g	[21]
		Cassava starch			-	After gelatinization, it has high viscosity but low stability and is used for thickening food.	15–35 m^2^/g	[22]
	Root and stem starch	Sweet potato starch	Irregular shape	-	-	It has a high viscosity after gelatinization and is often used for thickening food.	10–30 m^2^/g	[23]
According to the structure	High amylose	High amylose content in corn	-	Direct links >50%	-	The molecules are closely arranged, heat-resistant, and used in degradable packaging materials.	5–20 m^2^/g	[12]
	High amylose	Cassava starch	-	Branched chains >80%	-	The branch structure hinders rearrangement. After gelatinization, it has high viscosity and good transparency, and is used as a food gelling agent.	20–40 m^2^/g	[13]
According to physical and chemical properties	Common starch	Potato starch	Large particles with a smooth surface	Direct links account for approximately 20%	58–65 °C	It is applicable to conventional food processing.	20–40 m^2^/g	[17]
	Waxy starch	Waxy corn starch	-	The direct chain is approximately 0%	-	The paste has high stability and strong anti-reflux property, and is used for frozen food.	20–40 m^2^/g	[18]
	Waxy starch	Green banana starch	-	The direct chain is approximately 20–25%	-	It is difficult to be decomposed by enzymes, has prebiotic functions, and is used in low glycemic index foods.	10–30 m^2^/g	[19]

### 2.2. Classification of Rice Starch: Ordinary and Special Varieties

**Figure 1:**
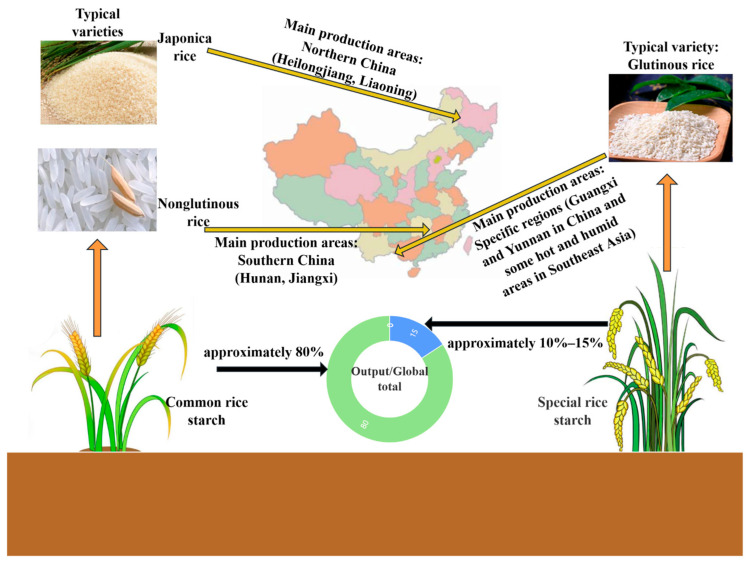
Rice starch production areas and yield map.

Rice starch can be categorized into ordinary and specialty types, based on variety genetics and cultivation conditions (Figure 1) [24].

Ordinary rice starch (e.g., from indica and japonica) typically has granules that are polygonal or spherical. With amylose content between 15 and 30%, it shows moderate viscosity and clarity postgelatinization but is prone to retrogradation during storage, limiting texture stability in foods [25].

Specialty rice starches, such as waxy rice starch from glutinous rice, consist almost entirely of amylopectin. Their granules, while similar in shape to ordinary starch, are smoother and gelatinize at lower temperatures (55–65 °C). The resulting pastes offer high viscosity, clarity, and strong resistance to retrogradation, making them suitable for high-quality frozen and instant food products [26].

### 2.3. Applications of Rice Starch

**Figure 2:**
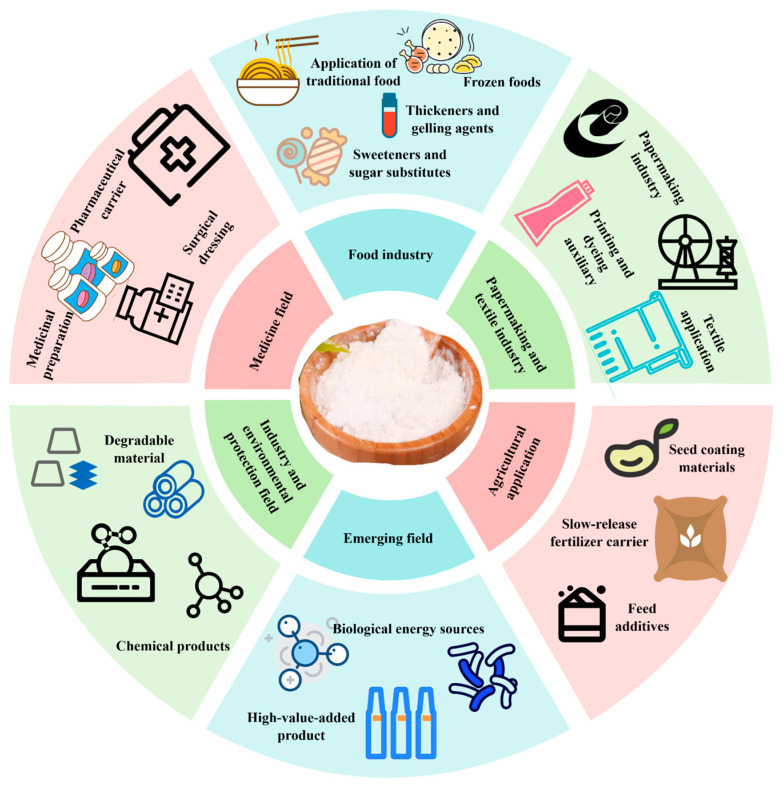
Application fields of rice starch.

Rice starch holds significant application value in both traditional industries and emerging fields due to its tunable linear-to-branched chain ratio and ease of chemical or physical modification (Figure 2) [27].

In the food industry, rice starch is widely used for its thickening and gelling properties in both traditional Asian foods and modern processed products [28]. For example, indica rice starch (with 20–25% amylose content) imparts elasticity and transparency to Vietnamese rice noodles through gelatinization, exhibiting a peak viscosity of up to 3000 BU [29]. Glutinous rice starch [30], which contains over 99% amylopectin, is a preferred gelling agent in frozen puddings and instant jellies due to its high viscosity (>5000 cP) and superior anti-retrogradation properties. In the development of functional foods, resistant rice starch (RS3 type) undergoes wet heat modification to increase its digestibility to over 60%, making it suitable for use in low glycemic index (GI) meal replacement powders [31]. High-amylose rice starch (e.g., mutant GM645, 34% amylose) can be fabricated into low-calorie rice paper for functional ingredient delivery due to its excellent film-forming ability [32].

In the pharmaceutical field, pregelatinized rice starch [33] (disintegration time < 5 min) has largely replaced traditional corn starch as a disintegrant in tablet formulations. Octenyl succinic anhydride (OSA)-modified rice starch enables the production of sustained-release capsules capable of achieving targeted drug delivery in simulated intestinal fluids, with release rates exceeding 90% within 6 h [34]. Furthermore, pH-responsive hydrogels formed by cross-linking rice starch with chitosan (cross-linking degree > 85%) demonstrate excellent biocompatibility in wound dressings, with clinical trials indicating a 25% reduction in the healing time of chronic ulcers [35].

In the papermaking and textile industries, rice starch is utilized as a sustainable sizing agent owing to its fine particle size (3–8 μm) and strong film-forming capability. Its application enhances the surface strength of paper (tensile strength increased by 20%) and reduces ink penetration during printing [36]. In textiles, glutinous rice starch slurry (sizing rate 8–12%) improves yarn abrasion resistance and reduces weaving breakage rates to below 1.5%, making it especially suitable for processing high-count cotton yarns [37].

In the industrial and environmental sectors, high-amylose rice starch (e.g., GM645) blended with polylactic acid (PLA) produces biodegradable films with a tensile strength of 18 MPa and a degradation rate exceeding 70% within 90 days in soil [38]. Oxidized rice starch (carboxyl content 0.8–1.2 mol%) is employed as an adhesive in corrugated paper production, increasing peel strength to 4.5 N/cm^2^—well above that of traditional corn starch adhesives (3.2 N/cm^2^) [39]. OSA-modified rice starch-based coatings (degree of substitution 0.03) can reduce VOC emissions by 60%, and have been applied in interior wall coatings for green buildings [40].

In emerging applications, rice starch is efficiently converted into bioethanol via simultaneous saccharification and fermentation, achieving a conversion efficiency greater than 90%. Thailand has established a commercial-scale rice husk starch ethanol plant with an annual production capacity of 100,000 tons [41]. Additionally, rice starch microcapsules produced via spray drying (encapsulation efficiency > 95%) are capable of encapsulating antioxidants such as astaxanthin, enabling sustained release over 12 h in cosmetic applications and significantly enhancing the stability of active ingredients [42].

Beyond the above-mentioned applications, rice starch holds promise as a sustainable bio-template, structure-directing agent, green reductant, and stabilizer in sol–gel synthesis. This low-temperature wet chemical process utilizes starch to fabricate high-value functional materials, such as mesoporous oxides and nanocomposites, for applications in catalysis, energy storage (e.g., battery electrodes), and biomedicine [43]. The renewable nature, cost-effectiveness, and unique structural properties (e.g., templating mesoporosity) of starch offer significant advantages in designing novel materials [44].

## 3. Driving Effect of Interfacial Properties on the Functional Enhancement of Rice Starch

Although emulsion technology has greatly improved the emulsifying performance, environmental adaptability, and functional diversity of rice starch through interfacial engineering, its practical application remains constrained by the intrinsic physicochemical limitations of native starch. This section systematically analyzes three core issues associated with natural rice starch, namely functional singularity, poor stability, and environmental sensitivity, and aims to provide a theoretical foundation for subsequent strategies to enhance its functionality.

### 3.1. Limitations of Rice Starch

The limitations of rice starch are primarily reflected in three aspects: limited functionality, poor stability, and sensitivity to environmental conditions [45]. Native rice starch chains lack hydrophobic groups, making them ineffective at adsorbing onto oil–water interfaces and thereby exhibiting poor emulsifying capacity. Moreover, amylose is prone to retrogradation at low temperatures, leading to gel hardening and water syneresis, which severely affect product shelf life [46]. In addition, extreme pH conditions, high temperatures, or elevated ionic strength can disrupt the granular structure of starch, further compromising its practical utility [7].

### 3.2. Functional Enhancement of Rice Starch via Emulsion Technology

**Figure 3:**
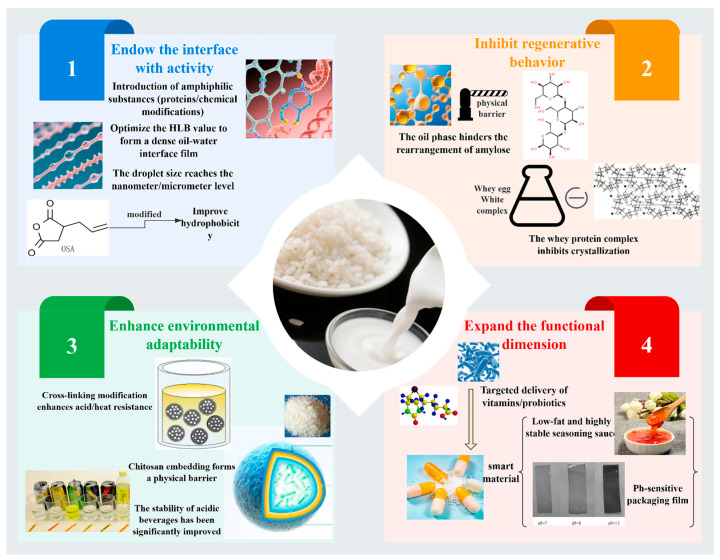
Schematic diagram of how emulsion technology helps upgrade the functionality of rice starch.

Emulsion technology offers a comprehensive approach to overcoming the functional limitations of rice starch by means of interfacial engineering and molecular structure modification. Core strategies include enhancing interfacial activity, inhibiting retrogradation, improving environmental adaptability, and expanding functional attributes (Figure 3). The specific implementation pathways are outlined below.

#### 3.2.1. Imparting Interfacial Activity to Rice Starch

**Figure 4:**
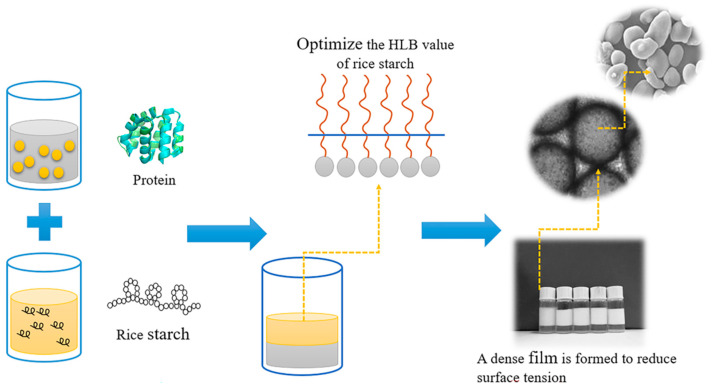
Mechanism diagram of emulsion interface stabilization mechanism.

By introducing amphiphilic substances (e.g., proteins) or through chemical modifications such as esterification and etherification, emulsion technology can optimize the hydrophilic–lipophilic balance (HLB) of rice starch, enabling stable adsorption at the oil–water interface and the formation of a compact interfacial film that reduces interfacial tension (Figure 4) [8]. For instance, rice starch modified with octenyl succinic anhydride (OSA) exhibits significantly enhanced hydrophobicity and emulsifying performance comparable to that of synthetic surfactants (e.g., Tween series), while offering superior biocompatibility [47]. This approach not only ensures uniform emulsion droplet size (ranging from nano- to microscale), but also reduces reliance on petroleum-based emulsifiers, aligning with green chemistry principles [48].

In a study by Jain et al. [49], modified rice starch was used to fabricate delivery systems such as water-in-oil emulsions and alginate beads for encapsulating lycopene, a hydrophobic compound with low chemical stability. When stored at 4–70 °C for 15 days, the lycopene degradation followed the order: solution > emulsion > alginate beads. Simulated gastrointestinal model experiments showed that the extent of lipid digestion was positively correlated with lycopene bioaccessibility. Lycopene bioaccessibility was significantly higher in the emulsion system (20.2%) compared to the beads (15.6%), while chemical stability was better in alginate beads (35.6%) than in emulsions (29.5%). These results demonstrate that modified rice starch is a promising food-grade material for developing delivery systems that enhance the chemical stability and controlled gastrointestinal release of lipophilic bioactives such as lycopene.

#### 3.2.2. Inhibition of Rice Starch Retrogradation

In emulsion systems, the oil phase acts as a physical barrier that hinders the rearrangement of linear rice starch molecular chains, thereby slowing the retrogradation process [50]. Simultaneously, complexes formed between interfacial stabilizers (such as whey protein) and starch molecules can further suppress crystallization [51]. For example, incorporating rice starch-based emulsions into frozen dough significantly enhances storage stability and reduces the need for anti-staling additives, thereby lowering production costs. However, high oil-phase ratios may lead to undesirably firm textures, thus necessitating additional technologies (e.g., enzymatic inhibition) to balance performance and cost-effectiveness [52].

Dun et al. [53] found that the addition of emulsions decreased the peak viscosity and gelatinization enthalpy of rice starch, limited granule swelling, and reduced the final gel strength. After 14 days of storage at 4 °C, the relative crystallinity of the rice starch gel decreased from 11.59% to 6.81%, resulting in reduced hardness and water mobility, and a denser, more uniform gel microstructure. These findings confirm that emulsions can effectively inhibit both short-term and long-term retrogradation of rice starch, supporting their application in starch-based food products.

#### 3.2.3. Enhance Environmental Adaptability

The resistance of rice starch to acidic and thermal environments can be enhanced through cross-linking modification or composite encapsulation (e.g., blending with chitosan). Additionally, the interfacial barrier formed in emulsions can protect starch structures from environmental stressors such as pH fluctuations and ionic strength [54]. For instance, cross-linked rice starch exhibits superior stability in acidic beverages and thermally processed foods, thereby reducing the need for auxiliary stabilizers and simplifying formulation design. However, it is important to note that cross-linking may compromise the biodegradability of starch, thus necessitating a trade-off between enhanced functionality and environmental sustainability [55].

Montoya Yepes et al. [56] encapsulated phenolic compounds from passion fruit seed extract using enzymatically acylated rice starch as the wall material, via both emulsion and spray-drying techniques. The encapsulation efficiencies exceeded 40–90%, respectively. The encapsulated system exhibited targeted release at alkaline pH and retained antioxidant activity for up to 8 months. These results demonstrate that modified rice starch, via cross-linking and composite encapsulation, enhances resistance to environmental and physiological stresses and holds promise for use in phenolic compound stabilization and herbal medicine delivery.

#### 3.2.4. Expanding Functional Applications

Emulsion technology opens new pathways for expanding the functional versatility of rice starch. By encapsulating functional ingredients (such as vitamins and probiotics), rice starch-based emulsions can serve as vehicles for targeted and controlled release, particularly in pharmaceutical applications [57]. Furthermore, the development of intelligent responsive materials, such as pH-sensitive packaging films based on Pickering emulsions, highlights the potential of rice starch in smart material design. For example, the successful development of low-fat, high-stability seasoning sauces demonstrates the high-value applications of rice starch-based emulsions in the food industry [58].

Kamwilaisak et al. [59] prepared rice starch nanoparticles (SNPs) via HCl hydrolysis and citric acid cross-linking and used them to stabilize sunflower oil-in-water Pickering emulsions containing curcumin. Under optimal hydrolysis conditions (2.2 M HCl, 6 days), the SNP yield was 21.87 ± 0.69%, with a crystallinity of 45.56%. The SNPs modified with 6 h citric acid cross-linking (SNP-M-6 h) had a water contact angle of 87.2°, and 3.0 wt% SNP-M-6 h effectively stabilized emulsions containing 30 wt% curcumin-loaded sunflower oil. The emulsions exhibited shear-thinning, pseudoplastic behavior, with a droplet size of 47.16 ± 4.22 μm, and remained highly stable after 5 weeks of storage. Curcumin release was pH-dependent—slower under acidic and faster under alkaline conditions—demonstrating potential for oral drug delivery applications.

## 4. Application Mode of Starch in Emulsion Systems

**Figure 5:**
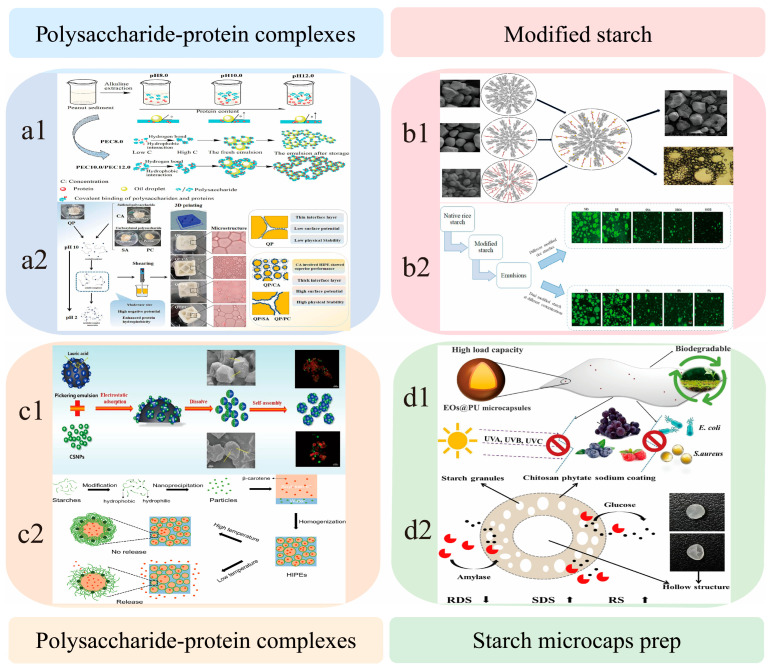
Application model of starch in emulsion system (Reproduced or adapted from [60,61,62,63,64,65,66,67,68,69,70,71], with permission from ELSEVIER Ltd., 2025.). (**a1**): Schematic of the preparation process for peanut protein sediment-based emulsions under an alkaline environment (pH 8.0–12.0). (**a2**): Mechanistic diagram illustrating the effect of PEC (polysaccharide–protein complex) concentration on the stability of the interfacial layer in peanut protein emulsions. (**b1**): Flowchart of the Pickering emulsion preparation process using lauric acid-modified starch particles. (**b2**): Schematic of the microstructure and stability maintenance of HIPEs (high internal phase emulsions) stabilized by starch-based CSNPs. (**c1**): Schematic of electrostatic adsorption, self-assembly, and multiscale characterization (SEM & fluorescence imaging) of lauric acid-modified Pickering emulsions with CSNPs. (**c2**): Flowchart of temperature-responsive HIPE preparation via starch hydrophobic modification and nanoprecipitation, illustrating β-carotene encapsulation and release mechanisms. (**d1**): Flowchart of the preparation process for starch–chitosan phytate composite-coated microcapsules. (**d2**): Schematic of the antibacterial properties of EOs@PU microcapsules and their amylase-responsive controlled release mechanism.

Building on the optimization of starch functionalities through interfacial engineering, this section explores four application modes of starch-based emulsions (Figure 5), with a focus on their practical potential in food and pharmaceutical fields. Notably, the nature of the protein component greatly influences the interfacial behavior of the complex. For instance, plant proteins such as pea or wheat gluten exhibit different solubility profiles and surface hydrophobicity compared to dairy proteins, which affect their compatibility and binding behavior with starch during emulsion formation.

### 4.1. Polysaccharide–Protein Complexes

In food emulsion systems, combining polysaccharides (e.g., starch) with proteins offers notable synergistic benefits. The interfacial stabilization mechanism leverages the hydrophobicity of proteins and the hydrophilicity of starch to form complexes via physical mixing or chemical cross-linking (e.g., whey protein–starch complexes) that maintain emulsion stability even under acidic conditions. This characteristic is particularly advantageous for quality control in fermented foods such as yogurt [72].

Further studies have demonstrated that dual treatments, such as ultrasonic processing combined with heat, significantly enhance the functional properties of soy protein–rice starch complexes (Table 2). Improvements in solubility, water retention, and foaming capacity are closely associated with intermolecular hydrogen bonding particularly short-range interactions between amino acids (e.g., glutamine and leucine) and hydroxyl groups on starch. These interactions broaden the functional applications of such complexes in emulsion systems.

While these complexes are attractive due to their use of natural, clean-label ingredients, their stability remains sensitive to environmental fluctuations in pH and temperature. Therefore, process optimization is essential to balance functionality and economic feasibility. Nonetheless, research on molecular interactions (e.g., protein complex formation), structural regulation (e.g., amylose content modulation), and processing technologies (e.g., ultrasound-assisted treatment) has provided a theoretical and technical foundation for the transition of polysaccharide–protein complexes from laboratory research to industrial applications. Compared to synthetic emulsifiers, protein–starch complexes offer advantages in clean-label formulation, biocompatibility, and biodegradability. Additionally, they can be tailored to meet the growing demand for allergen-free or plant-based emulsions in the food and nutraceutical sectors. However, batch-to-batch variability and scalability of production remain critical barriers. Current research efforts should therefore focus not only on understanding molecular mechanisms but also on process standardization and shelf-life optimization to enable industrial translation.

Hayashi et al. [73] reported that in the ss1 L/ss2a L/ss3a mutant rice line, the activities of three major starch synthases were reduced to 25–30% of wild-type levels. Starch content in grains dropped to 55%, while the apparent amylose content increased to 34%. Protein analyses indicated altered enzymatic interactions and the formation of new active complexes, even with reduced starch synthase isozyme levels. The mutant exhibited fewer branched chains and more linear chains, revealing a connection between starch structure and protein complex formation.

Thirunavookarasu et al. [74] investigated the effect of ultrasonic cavitation on soy protein–rice starch complex formation. At 60% ultrasonic amplitude (450 W) combined with heat treatment, the resulting complex exhibited significantly improved solubility, water retention, and foaming capacity compared to untreated soy protein isolate. Crystallinity of the complex was preserved. Molecular docking using 11S protein (1FXZ) confirmed short-range hydrogen bonding between glutamine and leucine residues and starch hydroxyl groups, resulting in a complex with excellent functional characteristics suitable for food applications. These findings reinforce the importance of short-range molecular interactions in stabilizing emulsions, particularly under stress conditions such as heating or acid exposure, which are common in food processing scenarios.

Hwang et al. [75] identified that rice endosperm starch phosphorylase (Pho1) and 4-α-glucanotransferase (Dpe1) assembled into a 1:1 molar ratio protein complex. This interaction was verified through pull-down assays, immunoprecipitation, chromatographic co-elution, and electrophoretic co-migration. The complex could utilize a broader range of substrates to synthesize larger maltooligosaccharides compared to the individual enzymes. Notably, the complex significantly enhanced Pho1′s substrate affinity at 30 °C and enabled it to act on transglycosylation products (G1 and G3), which it otherwise could not catalyze independently.

Chen et al. [76] compared gene expression and protein interactions in white-core (GM645) and glutinous (GM077) mutants derived from high-amylose Indica rice Guangludwarf 4. During seed development, key starch biosynthesis genes such as AGPS1 and GBSSI were significantly downregulated in GM645, while AGPL2 was upregulated in GM077. Immunoprecipitation analysis specifically detected interactions between key enzymes (including SSI-SSIIa, SSIIa-SBEIIb, and SBEIIb-PUL), revealing distinct alterations in the mutants: for example, the SSI-SSIIa interaction was weak/absent in GM077 and reduced in GM645, while the SBEIIb-PUL interaction was notably reduced. These findings provide insight into the role of protein complexes in starch biosynthesis, particularly in *Indica* rice.

Moreover, the degree of starch branching and amylose-to-amylopectin ratio are critical determinants of emulsion performance when complexed with proteins. Higher amylose content tends to promote linear interactions and network formation, contributing to emulsion viscosity and gelation. In contrast, highly branched amylopectin may reduce the strength of protein–starch interactions due to steric hindrance. Understanding these structure–function relationships enables the rational design of emulsifying agents by tailoring starch structural features in combination with specific proteins.

### 4.2. Modified Starch

Chemical and physical modifications represent key strategies to enhance the interfacial activity of starch in emulsion systems. Chemical modifications—such as esterification and etherification—introduce hydrophobic functional groups, thereby improving emulsifying capacity. Physical modifications, including wet heat treatment, alter molecular conformation and enhance functional adaptability. Among these, octenyl succinic anhydride (OSA)-modified starch is particularly notable for its emulsifying performance, which approaches that of synthetic emulsifiers. OSA-modified starch also demonstrates superior resistance to acid and heat, making it highly desirable for food applications. However, its industrial-scale production requires stringent control over residual chemicals to meet safety standards [77]. Compared to chemical modification, physical approaches offer a more sustainable and consumer-acceptable route, especially in clean-label product development. However, their relatively limited ability to alter molecular structures results in lower interfacial activity. In contrast, chemical modification achieves higher emulsification efficiency but raises concerns related to chemical residues, regulatory approval, and consumer perception. Therefore, the choice of modification strategy often hinges on the specific product requirements, regulatory constraints, and target consumer expectations.

**Figure 6:**
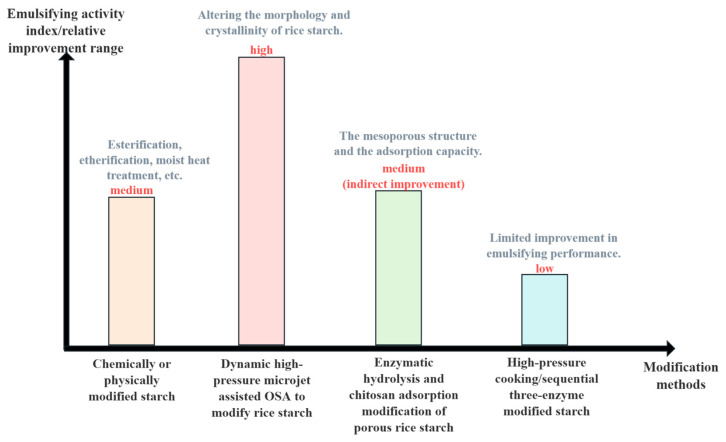
Effects of different modification methods on emulsification performance.

By contrast, physical modification methods, though more cost-effective and environmentally friendly, often result in more modest performance improvements. Therefore, they are commonly combined with other techniques to meet practical application requirements (Figure 6).

Li et al. [78] investigated the chemical modification of rice starch using dynamic high-pressure microfluidization (DHPM) in conjunction with OSA treatment. DHPM pretreatment altered the morphology and crystallinity of the starch, increasing its degree of substitution. Compared to OSA-modified rice starch without DHPM, the pretreated samples exhibited higher peak viscosity, lower gelatinization temperature, and significantly enhanced emulsifying activity and stability. These findings highlight DHPM as a promising tool to modulate the physicochemical properties of starch, thereby improving its reactivity and effectiveness in chemical modification processes. These findings underscore the importance of interfacial engineering in starch modification. By altering particle morphology, charge distribution, and hydrophilic–hydrophobic balance, such modifications enable starch molecules to more effectively migrate to and stabilize the oil–water interface, thereby enhancing emulsion performance.

Jiang et al. [79] developed a porous rice starch by enzymatic hydrolysis, followed by surface modification with chitosan. The resulting starch featured a mesoporous structure with a specific surface area of 1.13 ± 0.05 m^2^/g and interacted with chitosan via intermolecular hydrogen bonding. Chitosan functionalization rendered the starch surface positively charged across a broad pH range (2–10). Enzymatic hydrolysis slightly reduced the particle size and apparent viscosity, while chitosan coating increased both the particle size and viscosity. Remarkably, the modified starch showed strong adsorption capacity for proanthocyanidins, capturing up to 96 ± 3% through chemical adsorption or strong surface complexation rather than simple mass transfer. This study demonstrates that chitosan-modified porous rice starch can serve as an efficient polyphenol adsorbent, with chitosan playing a crucial role in enhancing adsorption performance. Beyond polyphenol adsorption, such chitosan–starch hybrid systems may also serve as multifunctional emulsifiers with antioxidant capacity, pH responsiveness, or controlled-release behavior, offering extended application possibilities in nutraceutical emulsions and delivery systems.

Li et al. [78] compared several starch modification strategies: high-pressure cooking alone (A-MS), high-pressure cooking combined with pullulanase treatment (A/PUL-MS), and a sequential three-enzyme modification (β-amylase → transglucosidase → pullulanase) following high-pressure cooking (A/STE-MS), using natural rice starch (NRS) as a control. The A/STE-MS sample exhibited a dense granule structure, with a high proportion of short chains (43.17%, DP ≤ 6). Apparent amylose contents were elevated in A/PUL-MS (38.39%) and A/STE-MS (33.78%), which also showed improved thermal stability. X-ray diffraction revealed distinct crystalline patterns: NRS had an A-type crystal structure, A/PUL-MS was B-type, and both A-MS and A/STE-MS showed V-type crystallinity. A/STE-MS further demonstrated high swelling power and solubility index, low apparent viscosity, and a reduced glycemic index (62.9), underscoring its potential in developing low-GI food products. These results suggest that enzyme-assisted high-pressure modification can significantly tailor rice starch structure and functionality, offering new opportunities for food formulation and industrial applications.

Despite significant progress in starch modification, several challenges remain. Achieving a balance between functionality, process scalability, and regulatory compliance is still a major concern. Future efforts may focus on developing hybrid modification methods, such as integrating physical, enzymatic, and mild chemical treatments, to produce high-performance, food-grade emulsifiers. Additionally, real-time characterization of interfacial behavior and long-term stability studies in complex food matrices are essential for industrial application.

### 4.3. Starch-Based Pickering Emulsion

Starch particles can be converted into solid emulsifiers through nano-sizing or hydrophobic modification, enabling their adsorption at the oil–water interface to form a robust physical barrier [80]. These Pickering emulsions, known as Pickering emulsions and stabilized exclusively by solid particles rather than molecular surfactants [81], eliminate the need for small-molecule emulsifiers, making them particularly attractive for natural food formulations. Additionally, the particle-based barrier provides excellent long-term stability, including resistance to Ostwald ripening, a thermodynamically driven process where smaller droplets shrink (due to higher solubility) and larger droplets grow over time, leading to emulsion instability [82]. However, precise control over particle size and surface wettability remains technically challenging, and large-scale production often requires advanced equipment such as high-pressure homogenizers.

Taghavi et al. [83] explored the use of modified rice starch particles as plant-based emulsifiers for mayonnaise-based Pickering emulsions. The mayonnaise formulation contained 50% (*w*/*w*) olive oil and 50% (*w*/*w*) lemon juice, with natural and modified rice starch added at concentrations of 200 and 400 mg starch/mL oil. Emulsification ability and stability were assessed over 1, 7, and 14 days. The results showed that low concentrations (200 mg/mL) of pregelatinized rice starch produced emulsions with an emulsion index of up to 100% and the smallest droplet size (17.0 ± 10.55 μm). All formulations displayed shear-thinning behavior. Emulsions stabilized with pregelatinized starch exhibited the highest viscosity and viscoelasticity (G′ > G″), along with superior storage stability. These findings suggest that pregelatinization is an effective strategy to enhance the functionality of rice starch in emulsified systems and represents a promising approach for developing clean-label mayonnaise alternatives.

Ji et al. [84] designed a ternary complex based on octenyl succinate-modified millet starch, chitosan hydrochloride, and epigallocatechin gallate to stabilize high-internal-phase Pickering emulsions. The complex was formed via electrostatic, hydrophobic, and hydrogen bonding interactions. At a mass ratio of 60:15:4, the complex exhibited the smallest particle size (277.6 nm), highest hydrophobicity (88.17°), and strong antioxidant activity. When used at concentrations ≥1% (*w*/*v*), it successfully stabilized high-internal-phase emulsions with excellent storage and centrifugal stability for up to 28 days. At 3.5% (*w*/*v*), the emulsion also functioned as a printable bio-ink with high 3D printing accuracy and shape retention. Such ternary complexes not only contribute to droplet stabilization via multipoint interactions but also endow emulsions with antioxidant and rheological functionalities, making them ideal for multifunctional delivery systems or next-generation food materials like printable bio-inks. This study demonstrates the potential of modified starch from underutilized sources such as millet in advanced emulsion technologies.

Gong et al. [85] investigated the effect of electric dry heating (EDH) and baking dry heating (RDH) on the performance of octenyl succinic anhydride (OSA)-modified rice starch in stabilizing water-in-oil Pickering emulsions. Heat treatments preserved starch granule morphology but reduced gelatinization temperature and enthalpy, while maintaining type A crystallinity. In vitro digestibility increased and relative crystallinity decreased from 26.73% to 21.74%. Emulsions stabilized by RDH-treated starch (180 °C) exhibited larger droplet volumes, whereas EDH-treated starch had more consistent performance. These results indicate that combining dry heat treatment with hydrophobic modification enhances the performance of starch-based Pickering emulsifiers and may serve as a practical strategy for industrial-scale applications.

Despite their promising performance, starch-based Pickering emulsions face technical challenges including poor redispersibility after drying, sensitivity to environmental changes, and batch-to-batch variability. Future research should focus on developing scalable particle engineering techniques, establishing structure–function databases, and exploring co-stabilization strategies (e.g., protein or polysaccharide blends) to improve formulation robustness across various food matrices.

### 4.4. Preparation of Starch-Based Microcapsules

**Figure 7:**
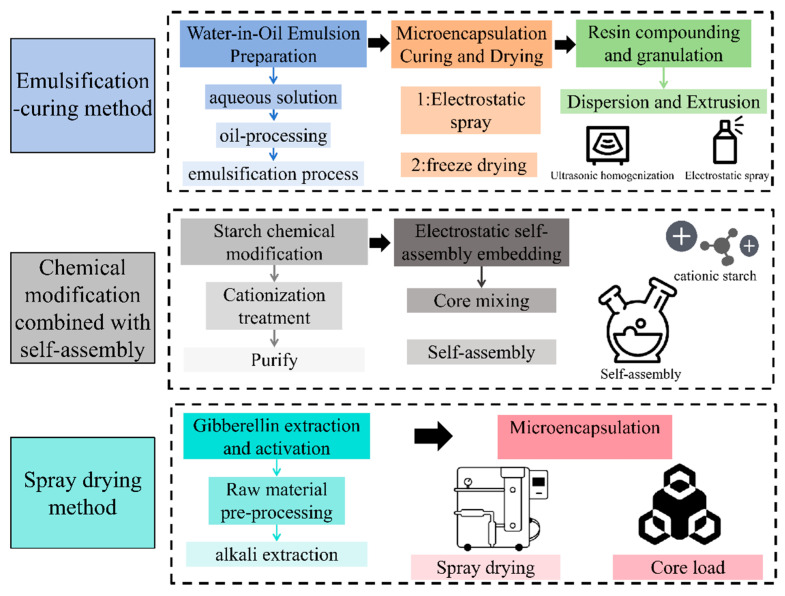
Microcapsule preparation process flow chart.

In starch-based microcapsules, interfacial stabilization during emulsification plays a key role in forming uniform droplets, which then undergo solidification to create protective matrices. The surface properties and interaction strength of starch determine the microcapsule’s structural integrity and release behavior under gastrointestinal or storage conditions. Starch is widely used as a wall material for encapsulating oil-soluble active ingredients (e.g., essential oils, probiotics) via emulsification–solidification techniques such as spray drying (Figure 7). This encapsulation significantly improves the stability and controlled release of the core material [86]. For instance, starch-based microcapsules can enable the targeted release of probiotics in the intestinal tract. While mechanical strength and moisture sensitivity still need optimization, starch microcapsules exhibit substantial potential for use in functional foods and drug delivery systems. Although industrial-scale production entails high energy consumption, the cost can be reduced through process optimization.

Wu et al. [87] developed curcumin-loaded microcapsules using porous rice starch combined with xanthan gum as the wall material. Different ratios were tested, and the microcapsules were characterized via SEM, XRD, DSC, and FTIR. All samples achieved encapsulation efficiencies exceeding 90%, with an average particle size of approximately 3.30 µm. Curcumin formed inclusion complexes rather than simple physical mixtures within the matrix. In simulated gastrointestinal environments, the release of curcumin followed first-order kinetics in gastric fluid (R^2^ > 0.974) and the Higuchi model in intestinal fluid (R^2^ > 0.969), demonstrating effective controlled release. These findings highlight the promising role of porous rice starch/xanthan gum composites in delivering bioactive compounds.

Espinosa-Solís et al. [88] prepared various modified starches, including phosphorylated waxy corn starch, OSA-modified corn starch, and OSA-modified rice starch, via reactive extrusion and evaluated their performance in stabilizing water-in-olive oil Pickering emulsions. Microfluidization and spray drying were employed to assess encapsulation efficiency and oil protection. OSA-modified rice starch exhibited the smallest particle size and the most negative ζ-potential, indicating superior emulsifying and digestibility-enhancing properties. Phosphorylated waxy corn starch, with its spray-drying compatibility, improved oil stability and lowered digestibility, suggesting its suitability as a microcapsule wall material.

Ashwar et al. [89] utilized resistant starch (RS4) derived from rice starch as an encapsulation agent for targeted probiotic delivery. The study focused on three probiotic strains—*Lactobacillus casei*, *L. brevis*, and *L. plantarum*. RS4 microcapsules achieved encapsulation efficiencies between 43.01% and 48.46%, with particle diameters ranging from 45.53 to 49.29 μm. FTIR revealed characteristic peaks of bacterial presence, and DSC confirmed excellent thermal stability. Encapsulated probiotics maintained high viability (>7 log CFU/g) under simulated gastrointestinal conditions and after two months of cold storage (4 °C). These results indicate that RS4 is a promising oral delivery carrier for probiotics, offering protection against harsh environmental stresses.

As natural, biodegradable, and functional carriers, starch-based microcapsules offer a green alternative for encapsulation in food and pharmaceutical industries. Future research could explore intelligent release systems responding to pH or enzymatic triggers, or integrate bioactive packaging functionalities for the real-time protection and delivery of sensitive compounds.

## 5. Conclusions and Outlook

### 5.1. Summary

This review highlights recent progress in starch-based emulsion systems, focusing on interfacial engineering and structural modification techniques to improve the functional properties of native starch. Modified starches, through chemical, physical, or enzymatic treatments, demonstrate improved emulsifying ability by enhancing their surface activity and interfacial film formation, which contributes to better emulsion stability. Such modifications can mitigate common issues in starch-based systems, including phase separation and aggregation during storage, although the direct impact on starch retrogradation requires further detailed investigation. Additionally, the integration of functional bioactive compounds into starch-stabilized emulsions shows promising applications in food, pharmaceutical sustained release, and active packaging. However, challenges such as potential chemical residues from modification processes, process scalability, and environmental compatibility remain critical areas for future research and development.

### 5.2. Future Prospects

Future research should emphasize the development of environmentally friendly modification techniques, particularly those involving physical or enzymatic methods, to minimize chemical residues. The integration of intelligent manufacturing technologies—such as machine learning for process parameter optimization—can improve both product consistency and production efficiency. Furthermore, interdisciplinary approaches will facilitate the application of starch-based emulsions in emerging sectors such as cosmetics and sustainable energy. For instance, the use of starch-based Pickering emulsions in biodiesel formulations presents a promising research avenue. In addition, conducting comprehensive life cycle assessments (LCAs) will offer scientific guidance for the sustainable development of these systems. With continuous technological innovation and system-level optimization, the industrialization and commercialization prospects of starch-based emulsions are expected to expand significantly in the near future.

## Figures and Tables

**Table 2:** Common starch modification case table.

Research Object	Research Methods	Conclusions and Application Values	References
Starch–protein (whey protein, soy protein) composite systems	Physical mixing or chemical cross-linking	Utilize the synergistic effect of the hydrophobicity of proteins and the hydrophilicity of starches to stabilize the interface, which has important application value in fermented foods such as yogurt. Process optimization is required to balance functionality and economy.	[72]
ss1 L/ss2a L/ss3a mutant rice	Analysis of the activities of three major starch synthases, starch content in grains, apparent amylose content, and protein analysis	Starch synthesis in the mutants was retained, with fewer branched chains and more linear chains, revealing the relationship between starch properties and protein complexes.	[73]
Soy protein–rice starch complexes	Ultrasonic treatment (60% amplitude, 450 W) combined with heat treatment, molecular docking study using 11S protein (1 FXZ)	The formed complexes have excellent functional properties, expanding their application range in the food industry.	[74]
Rice endosperm starch phosphorylase (Pho1) and disproportionase (Dpe1)	Pull-down experiments, immunoprecipitation, chromatographic co-elution, electrophoretic co-migration, etc.	Verified the formation of the Pho1—Dpe1 complex and demonstrated the advantages of this complex in substrate utilization and catalytic reactions.	[75]
White-core (GM645) and waxy (GM077) mutants of high-amylose indica rice Guangluai 4	Determination of gene expression profiles and immunoprecipitation analysis	The protein–protein interactions in starch biosynthesis in mutants have changed, providing insights into the mechanism of starch biosynthesis, especially in indica rice.	[76]

## Data Availability

No new data were created or analyzed in this study. Data sharing is not applicable to this article.
